# Robotic versus laparoscopic gastrectomy for gastric cancer: a systematic review and meta-analysis

**DOI:** 10.1186/s12957-020-02080-7

**Published:** 2020-11-24

**Authors:** Jianglei Ma, Xiaoyao Li, Shifu Zhao, Ruifu Zhang, Dejun Yang

**Affiliations:** 1 Student of the Third Brigade, College of Basic Medical Sciences, Naval Medical University, No. 800 Xiangyin Road, Yangpu District, Shanghai, 200433 China; 2 Department of Gastrointestinal Surgery, Changzheng Hospital, Naval Medical University, No. 415 Fengyang Road, Huangpu District, Shanghai, 200003 China

**Keywords:** Robotic, Laparoscopic, Gastrectomy, Gastric cancer, Meta-analysis

## Abstract

**Background:**

To date, robotic surgery has been widely used worldwide. We conducted a systematic review and meta-analysis to evaluate short-term and long-term outcomes of robotic gastrectomy (RG) in gastric cancer patients to determine whether RG can replace laparoscopic gastrectomy (LG).

**Methods:**

The Preferred Reporting Items for Systematic Reviews and Meta-Analyses (PRISMA) statement was applied to perform the study. Pubmed, Cochrane Library, WanFang, China National Knowledge Infrastructure (CNKI), and VIP databases were comprehensively searched for studies published before May 2020 that compared RG with LG. Next, two independent reviewers conducted literature screening and data extraction. The quality of the literature was assessed using the Newcastle-Ottawa Scale (NOS), and the data analyzed using the Review Manager 5.3 software. Random effects or fixed effects models were applied according to heterogeneity.

**Results:**

A total of 19 studies including 7275 patients were included in the meta-analyses, of which 4598 patients were in the LG group and 2677 in the RG group. Compared with LG, RG was associated with longer operative time (WMD = −32.96, 95% CI −42.08 ~ −23.84, *P* < 0.001), less blood loss (WMD = 28.66, 95% CI 18.59 ~ 38.73, *P* < 0.001), and shorter time to first flatus (WMD = 0.16 95% CI 0.06 ~ 0.27, *P* = 0.003). There was no significant difference between RG and LG in terms of the hospital stay (WMD = 0.23, 95% CI −0.53 ~ 0.98, *P* = 0.560), overall postoperative complication (OR = 1.07, 95% CI 0.91 ~ 1.25, *P* = 0.430), mortality (OR = 0.67, 95% CI 0.24 ~ 1.90, *P* = 0.450), the number of harvested lymph nodes (WMD = −0.96, 95% CI −2.12 ~ 0.20, *P* = 0.100), proximal resection margin (WMD = −0.10, 95% CI −0.29 ~ 0.09, *P* = 0.300), and distal resection margin (WMD = 0.15, 95% CI −0.21 ~ 0.52, *P* = 0.410). No significant differences were found between the two treatments in overall survival (OS) (HR = 0.95, 95% CI 0.76 ~ 1.18, *P* = 0.640), recurrence-free survival (RFS) (HR = 0.91, 95% CI 0.69 ~ 1.21, *P* = 0.530), and recurrence rate (OR = 0.90, 95% CI 0.67 ~ 1.21, *P* = 0.500).

**Conclusions:**

The results of this study suggested that RG is as acceptable as LG in terms of short-term and long-term outcomes. RG can be performed as effectively and safely as LG. Moreover, more randomized controlled trials comparing the two techniques with rigorous study designs are still essential to evaluate the value of the robotic surgery for gastric cancer.

**Supplementary Information:**

**Supplementary information** accompanies this paper at 10.1186/s12957-020-02080-7.

## Introduction

At present, gastric cancer is still a serious threat to human health and it is the third leading cause of cancer death and the fifth most commonly diagnosed cancer in the world [1]. Surgical resection is considered to be the gold standard of treatment for gastric cancer and open gastrectomy with lymphadenectomy takes a dominant position in the treatment of gastric cancer. Since Kitano et al. [2] reported firstly LG for gastric cancer in 1994, LG has been gradually spread worldwide.

Minimally invasive surgery (MIS) represents a new trend for its unique features. In recent years, LG has been recognized for its advantages of MIS in the treatment of gastric cancer, such as less blood loss, reduced invasiveness, less postoperative pain, earlier recovery of intestinal function, shorter hospital stay, and less complication [3–9]. Clinical trials comparing laparoscopic with open surgery have shown that laparoscopic radical gastrectomy has the same long-term effects as open radical gastrectomy [10–12]. However, conventional laparoscopic surgery has also limitations of itself, including two-dimensional images, decreased sense of touch, amplification of hand tremor, lack of flexibility, and limited ranges of instrument movement. Besides, LG causes more physical stress and requires a long learning curve for surgeons, especially in lymph node dissection [13].

Recently, robot-assisted surgery, an emerging technology, has been used to overcome the technical drawbacks of conventional laparoscopic surgery. Advantages of robot-assisted surgery include high definition 3-D stereo video, convenient movements of the robotic arm, tremor suppression, and stable picture [14–16]. Application of the Da Vinci robotic surgical system has unlocked a new era of MIS, and it has been widely used in cardiovascular, urinary tract, hepatobiliary, and gynecological surgery [17]. Since Hashizume et al. [18] reported the first RG in 2002, studies on RG have been widely reported.

Many studies have reported the safety and feasibility of RG, which is meaningful in highlighting the status of RG in the treatment of gastric cancer. However, these studies included small sample size, a single institution design and different appraise system of complications, which limited them to conclude objective result. Therefore, there is no clear conclusion whether RG can achieve an equal or even better surgical effect to LG. We conducted this systematic review and meta-analysis to explore and compare the clinical efficacy of RG and LG.

## Methods

### Search strategy

The present study strictly complied with the relevant requirements of the PRISMA guidelines and completed the PRISMA checklist [19]. A systematic literature search was performed in Pubmed, Cochrane Library, WanFang, CNKI, and VIP for studies published before May 2020 that compared RG with LG, using the following searching terms: gastric cancer, gastric carcinoma, laparoscopic, robotic, and gastrectomy. In addition, the references of all relevant articles were also searched to find the additional literature. Only the studies in Chinese and English were included.

### Inclusion criteria and exclusion criteria

Included studies must meet the following criteria: (1) clinical research comparing RG with LG for patients with gastric cancer; (2) full-text article containing necessary data for statistical analysis, or including at least one of the following clinical outcomes: estimated blood loss, time to flatus, retrieved lymph nodes, operative time, length of hospital stay, proximal and distal margin distance, complications, mortality, OS, RFS, and recurrence rate; (3) if the same authors or center reported two or more studies, the most recent publication, the larger scale number publication or high-quality publication were included. If 2 or more studies included totally different patients from the same center, we still analyzed the datum from those studies.

Articles were excluded if they included any of the following criteria: (1) letters, review articles, conference reports, comments, case reports, and animal experimental studies; (2) articles including non-gastric cancer cases such as gastrointestinal stromal tumors, or benign gastric diseases; (3) articles without necessary data for statistical analysis.

### Data extraction and quality assessment of included studies

Two authors independently and carefully reviewed and extracted the effective data from all included studies according to the inclusion and exclusion criteria, and checked the results again. If there was a disagreement, the controversial results were resolved by further discussion, and a final decision was made. The following data were collected from each study: first author, publication year, country, study design, sample size (RG group and LG group), age, body mass index (BMI), extent of resection, estimated blood loss (EBL), time to flatus, retrieved lymph nodes, operative time, length of hospital stay, proximal and distal margin distance, complications, mortality, OS, RFS, and recurrence rate. If the research offered medians and ranges, the means and standard deviations (SDs) were estimated as described by Hozo et al. [20]. The NOS was used to estimate the quality of the included studies (http://www.ohri.ca/programs/clinical_epidemiology/oxford.asp). Scores range from 0 to 9 stars: studies with a score higher than or equal to 7 were considered to be high-quality and included in the meta-analysis, although it was generally believed that studies with a score of 6 or more were high quality.

### Statistical analysis

The meta-analysis was performed by using the Review Manager 5.3 software (Cochrane Collaboration, Oxford, UK). Continuous variables were assessed using weighted mean difference (WMD) with a 95% confidence interval (CI) and dichotomous variables using odds ratios (OR) with a 95% CI. The survival data, such as OS and RFS, was assessed using the hazard ratios (HR) and a 95% CI. The *I*^*2*^ statistics was utilized to evaluate the heterogeneity. *I*^*2*^ < 25%, 25% ≤ *I*^*2*^ ≤ 50%, and *I*^*2*^ > 50% were regarded as low, moderate, and high heterogeneity. If the test of heterogeneity was high (*I*^*2*^ > 50% or *P* < 0.05), a random-effect model was adopted. Otherwise, we used a fix effect model. Funnel plot was utilized to evaluate the potential publication of bias according to the overall complication. *P* < 0.05 was considered to be statistically significant.

## Results

### Selected studies

**Fig. 1:**
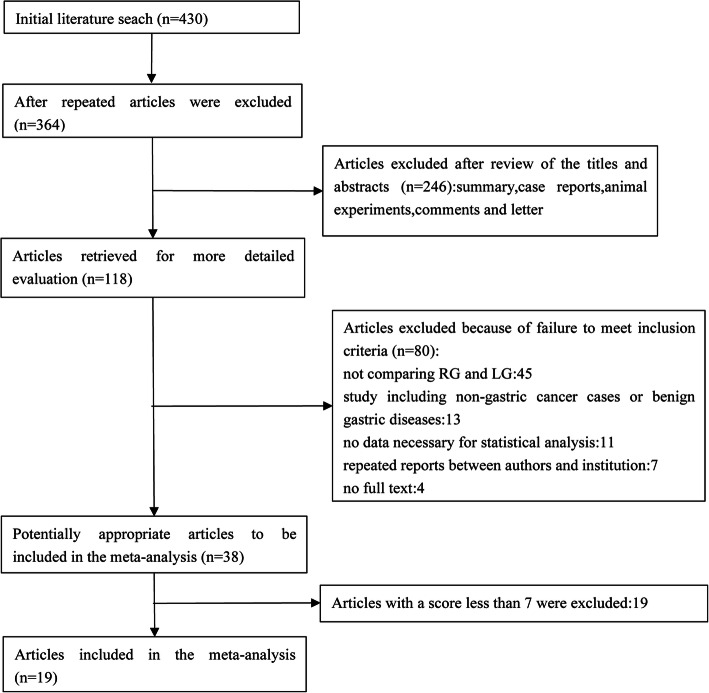
Flow chart of literature search strategies

A total of 430 potential articles, which were published before May 2020, were retrieved from our databases. After removing 66 duplicates, 246 studies excluded by carefully reading the titles and abstracts because it was a review, letter, conference report, comment, case report, or animal experimental study. One hundred eighteen potential articles were thoroughly evaluated through full-text articles, and finally, a total of 19 retrospective studies were included in the final meta-analysis according to inclusion and exclusion criteria [21–39]. A flow diagram of the search strategies, which includes reasons for the exclusion of studies, is shown in Fig. 1.

### Study characteristics and quality

Nineteen studies with a total of 7275 patients, of which 4598 patients were in the LG group and 2677 in the RG group, were involved. Fourteen of the included studies were published in English [23–30, 33–38], and 5 published in Chinese [21, 22, 31, 32, 39]. Among the 19 studies, 13 were from China [21–24, 29, 31–33, 35–39], 4 from Korea [25–27, 30], and 2 from Japan [28, 34]. The basic characteristics of the included studies are listed in Table 1. The evaluation of quality according to the NOS is shown in Table 2. NOS shows that 6 out of the 19 studies observed had 9 stars [24–26, 32, 34, 35], 2 had 8 stars [27, 33], and 11 had 7 stars [21–23, 28–31, 36–39].

**Table 1:** Main characteristics of studies included in the meta-analysis

Study	Year	Country	Design	Study period	Group	Cases	Age	BMI (kg/m^2^)	Surgical extension
Zhang [21]	2012	China	R	2009-2011	RG	97	56.1 ± 5.8	22.5 ± 3.6	D, P, T
					LG	70	54.8 ± 4.9	21.7 ± 2.1	
Liu [22]	2014	China	R	2012-2013	RG	100	66.4 ± 5.7	22.7 ± 1.8	D, P, T
					LG	100	67.8 ± 4.8	23.1 ± 1.2	
Huang [23]	2014	China	R	2008-2014	RG	72	67.7 ± 15.1	24.1 ± 3.3	D, T
					LG	73	66 ± 13.5	24.2 ± 3.3	
Zhou [24]	2014	China	R	2010-2013	RG	120	54.7 ± 10.1	21.6 ± 2.8	D,P,T
					LG	394	55.6 ± 11.8	21.7 ± 2.6	
Son T [25]	2014	Korea	R	2003-2010	RG	51	55.3 ± 12.2	22.7 ± 2.9	T
					LG	58	58.8 ± 12.2	23.2 ± 3.3	
Han [26]	2015	Korea	R	2008-2013	RG	68	50.6 ± 8.3	22.7 ± 2.4	PPG
					LG	68	49.8 ± 11.5	22.8 ± 3	
Lee [27]	2015	Korea	R	2003-2010	RG	133	53.6 ± 13.2	23.2 ± 2.7	D
					LG	267	59.2 ± 11.7	23.7 ± 2.8	
Suda [28]	2016	Japan	R	2009-2012	RG	88	63.5 ± 15.0	22.6 ± 4.6	D, T
					LG	438	64.0 ± 15.8	23.1 ± 6.4	
Shen [29]	2016	China	R	2011-2014	RG	93	56.8 ± 10.5	24.3 ± 3.3	D, T
					LG	330	57.9 ± 11.5	23.8 ± 3.6	
Hong [30]	2016	Korea	R	2008-2015	RG	232	53.7 ± 11.5	23.8 ± 3.3	D, P
					LG	232	55.0 ± 13.0	23.8 ± 3.0	
La n[31]	2017	China	R	2014-2016	RG	196	59.0 ± 11.6	23.6 ± 4.6	D, P, T
					LG	673	59.0 ± 11.6	23.5 ± 4.5	
Zhang [32]	2018	China	R	2011-2013	RG	70	58.0 ± 9.8	24.2 ± 3.4	D, P, T
					LG	70	56.9 ± 12.1	23.2 ± 2.9	
Li [33]	2018	China	R	2013-2017	RG	112	55.6 ± 11.3	23.6 ± 2.9	D, T
					LG	112	56.1 ± 11.1	23.6 ± 3.0	
Obama [34]	2018	Japan	R	2005-2009	RG	311	54.5 ± 12.6	23.6 ± 3.1	D, T
					LG	311	54.8 ± 12.0	23.2 ± 2.8	
Gao [35]	2019	China	R	2011-2014	RG	163	60.27 ± 10.50	23.77 ± 3.11	D, T
					LG	163	59.88 ± 11.72	23.25 ± 3.26	
Sun [36]	2019	China	R	2016-2018	RG	33	55.6 ± 10.3	22.38 ± 3.03	D, T
					LG	88	54.7 ± 10.9	22.59 ± 2.95	
Ye [37]	2020	China	R	2014-2019	RG	285	57.1 ± 8.3	24.4 ± 2.3	D
					LG	285	57.0 ± 8.6	24.5 ± 2.2	
Kong [38]	2020	China	R	2014-2017	RG	266	58.68 ± 10.54	24.23 ± 3.06	D, P, T
					LG	532	58.92 ± 9.82	24.25 ± 3.34	
Cui [39]	2020	China	R	2016-2019	RG	187	59.0 ± 10.5	24.1 ± 3.0	D
					LG	334	57.2 ± 11.9	23.8 ± 3.4	

*R* retrospectively collected data *D* distal gastrectomy, *P* proximal gastrectomy, *T* total gastrectomy, *PPG* pylorus-preserving gastrectomy, *BMI* body mass index, *LG* laparoscopic gastrectomy, *RG* robotic gastrectomy

**Table 2:** Assessment of the quality of the studies based on the NOS

Study	Selection (out of 4)				Comparability (out of 2)	Outcomes (out of 3)			Total (out of 9)
	(1)	(2)	(3)	(4)		(5)	(6)	(7)	
Zhang [21]	*	*	*	*	**	*			7
Liu [22]	*	*	*	*	**	*			7
Huang [23]	*	*	*	*	**	*			7
Zhou [24]	*	*	*	*	**	*	*	*	9
Son T [25]	*	*	*	*	**	*	*	*	9
Han [26]	*	*	*	*	**	*	*	*	9
Lee [27]	*	*	*	*	*	*	*	*	8
Suda [28]	*	*	*	*	*	*		*	7
Shen [29]	*	*	*	*	**	*			7
Hong [30]	*	*	*	*	**	*			7
Lan [31]	*	*	*	*	**	*			7
Zhang [32]	*	*	*	*	**	*	*	*	9
Li [33]	*	*	*	*	**	*	*		8
Obama [34]	*	*	*	*	**	*	*	*	9
Gao [35]	*	*	*	*	**	*	*	*	9
Sun [36]	*	*	*	*	**	*			7
Ye [37]	*	*	*	*	**	*			7
Kong [38]	*	*	*	*	**	*			7
Cui [39]	*	*	*	*	**	*			7

(1) Representativeness of the exposed cohort, (2) selection of the non-exposed cohort, (3) ascertainment of exposure, (4) demonstration that outcome of interest was not present at start of study, (5) assessment of outcome, (6) was follow-up long enough for outcomes to occur, (7) adequacy of follow-up of cohorts

### Short-term outcomes

**Fig. 2:**
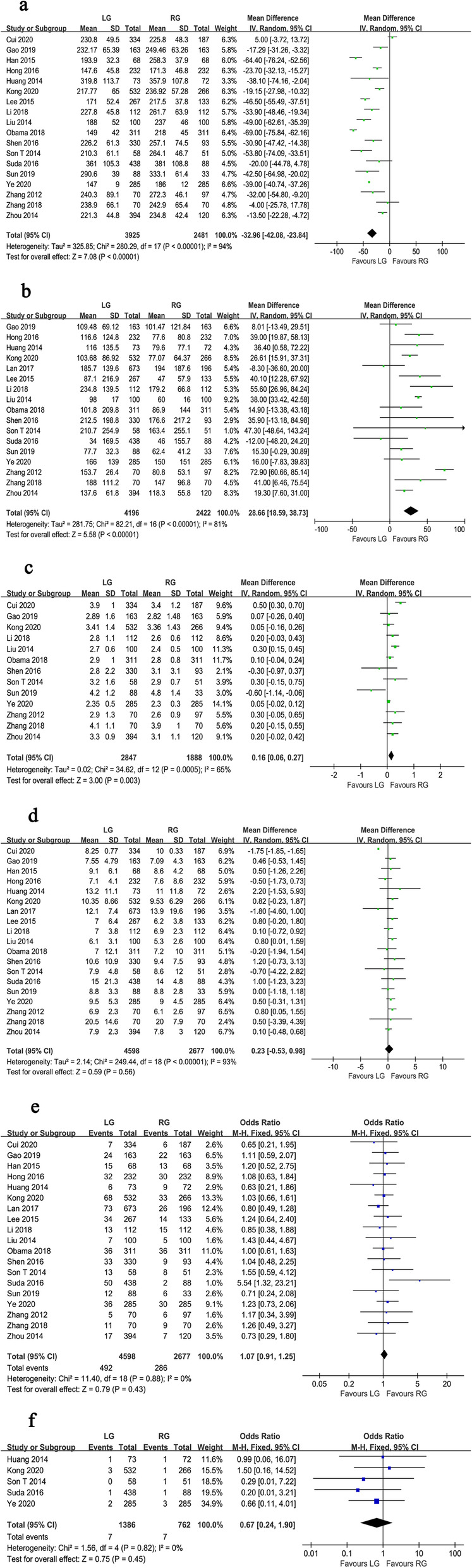
Forest plot of the meta-analysis for intraoperative and postoperative parameters. **a** Operation time. **b** Estimated blood loss. **c** Time to first flatus. **d** Length of hospital stay. **e** Overall postoperative complications. **f** Mortality

**Fig. 3:**
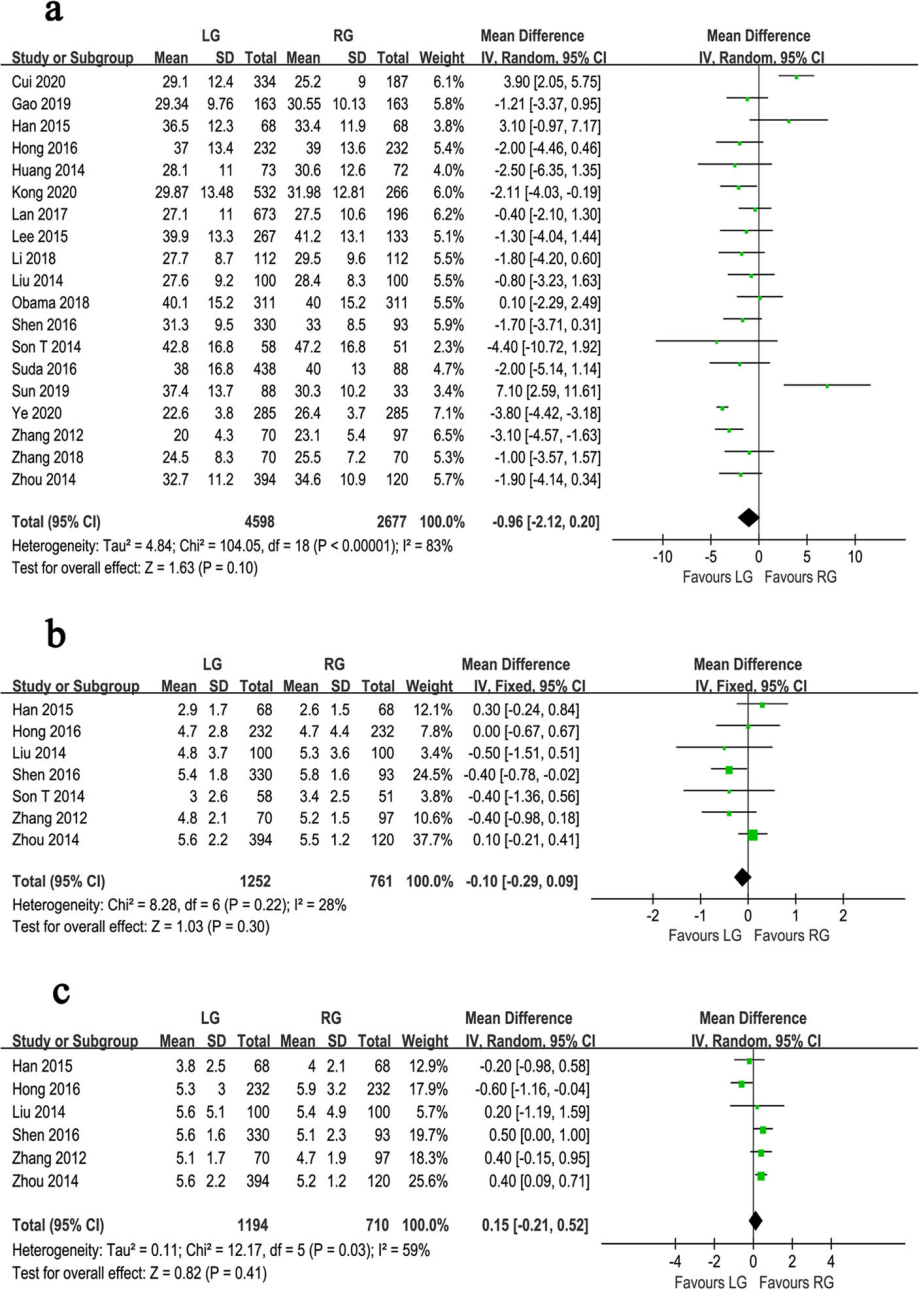
Forest plot of the meta-analysis for pathology details. **a** Number of retrieved lymph nodes. **b** Proximal margin distances. **c** Distal margin distance

**Fig. 4:**
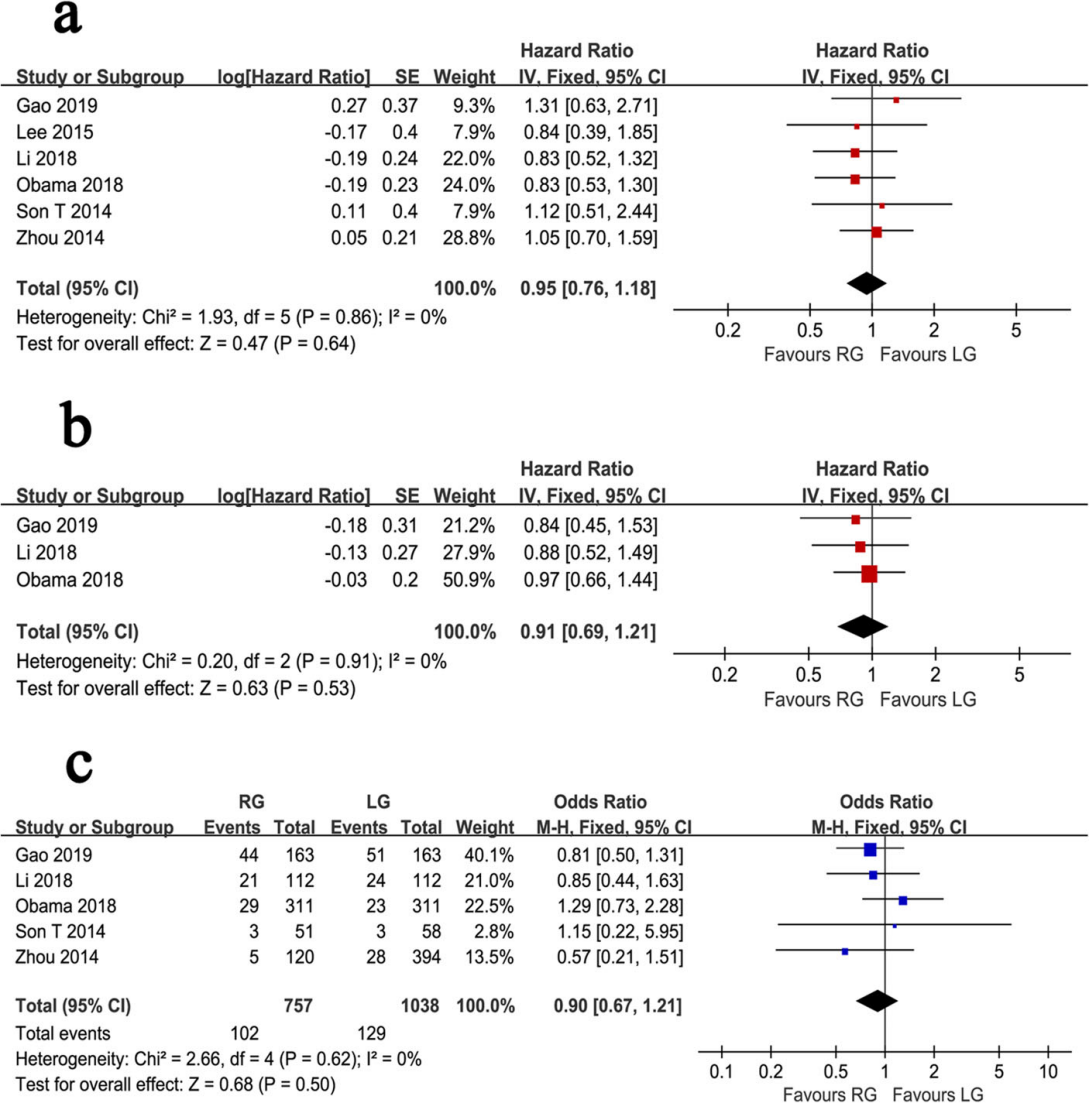
Forest plot of the meta-analysis for survival outcomes. **a** Overall survival. **b** Relapse-free survival. **c** Recurrence rate

Figs. 2, 3, 4, and Table 3 show the results of meta-analysis for short-term and long-term outcomes. Eighteen studies reported the operative time. Because there was significant heterogeneity between 18 studies (*I*^*2*^ = 94%, *P* < 0.001), a random effect model was adopted. Meta-analysis revealed that the operative time was longer for RG than for LG (WMD = −32.96, 95% CI −42.08 ~ −23.84, *P* < 0.001) (Fig. 2a). The EBL was reported in 17 studies. Because of significant heterogeneity (*I*^*2*^ = 81%, *P* < 0.001), a random-effect model was used. The meta-analysis showed that the EBL was lower in RG than LG (WMD = 28.66, 95% CI 18.59 ~ 38.73, *P* < 0.001) (Fig. 2b).

**Table 3:** Results of the meta-analysis

Outcomes	No. of studies	Sample size		Heterogeneity		Overall effect size	95% CI of overall effect	*P* value
		LG	RG	*I*^2^ (%)	*P* value			
Operation time (min)	18	3925	2481	94	< 0.001	WMD = −32.96	−42.08 ~ −23.84	< 0.001
Estimated blood loss (mL)	17	4196	2422	81	< 0.001	WMD = 28.66	18.59 ~ 38.73	< 0.001
Retrieved lymph nodes	19	4598	2677	83	< 0.001	WMD = −0.96	−2.12 ~ 0.20	0.100
Proximal margin (cm)	7	1252	761	28	0.220	WMD = −0.10	−0.29 ~ 0.09	0.300
Distal margin (cm)	6	1194	710	59	0.030	WMD = 0.15	−0.21 ~ 0.52	0.410
Time to first flatus (days)	13	2847	1888	65	< 0.001	WMD = 0.16	0.06 ~ 0.27	0.003
Hospital stay (days)	19	4598	2677	93	< 0.001	WMD = 0.23	−0.53 ~ 0.98	0.560
Overall complications	19	4598	2677	0	0.880	OR = 1.07	0.91 ~ 1.25	0.430
Mortality	5	1386	762	0	0.820	OR = 0.67	0.24 ~ 1.90	0.450
Overall survival	6	1498	890	0	0.860	HR = 0.95	0.76 ~ 1.18	0.640
Recurrence-free survival	3	586	586	0	0.910	HR = 0.91	0.69 ~ 1.21	0.530
Recurrence rate	5	1038	757	0	0.620	OR = 0.90	0.67 ~ 1.21	0.500

Pooled analysis showed that the number of days to first flatus of RG was shorter than LG, with a high heterogeneity (WMD = 0.16, 95% CI 0.06 ~ 0.27, *P* = 0.003, *I*^*2*^ = 65%) (Fig. 2c). All studies reported the days of hospital stay. A random effect model was used because of significant heterogeneity (*I*^*2*^ = 93%, *P* < 0.001). The pooled results showed no difference in hospital stay between the RG and LG groups (WMD = 0.23, 95% CI −0.53 ~ 0.98, *P* = 0.560) (Fig. 2d). All 19 studies presented the overall postoperative complication. Analysis of the index revealed no significant difference between the groups of RG and LG (OR = 1.07, 95% CI 0.91 ~ 1.25, *P* = 0.430) (Fig. 2e). The pooled result was measured using fixed effects models due to the lack of significant heterogeneity (*I*^*2*^ = 0%, *P* = 0.880). Moreover, 5 studies, with a total of 2148 gastric cancer patients, reported mortality. Pooled analysis showed no significant heterogeneity (*I*^*2*^ = 0%, *P* = 0.820) using a fixed effects model. Although no significant difference could be found in mortality between the two techniques, the pooled result revealed that LG group had a higher mortality than RG group (OR = 0.67, 95% CI 0.24 ~ 1.90, *P* = 0.450) (Fig. 2f).

All of the included studies reported the number of harvested lymph nodes. There was a significant heterogeneity, so a random effect model was adopted (*I*^*2*^ = 83%, *P* < 0.001). Analysis of the index revealed that harvested lymph nodes were similar between the groups of RG and LG (WMD = −0.96, 95% CI −2.12 ~ 0.20, *P* = 0.100) (Fig. 3a). Seven studies reported the proximal margin and a fixed effects model was adopted because no significant heterogeneity was observed (*I*^*2*^ = 28%, *P* = 0.220). The proximal margin was not significantly different between the two groups (WMD = −0.10, 95% CI −0.29 ~ 0.09, *P* = 0.300) (Fig. 3b). In terms of the distal margin, the difference between the two groups was not also significant (WMD = 0.15, 95% CI −0.21 ~ 0.52, *P* = 0.410), but the heterogeneity was significant (*I*^*2*^ = 59%, *P* = 0.030) (Fig. 3c).

### Long-term outcomes

The OS outcomes were recorded in 6 studies. Pooled analysis indicated no significant difference between the two techniques (HR = 0.95, 95% CI 0.76 ~ 1.18, *P* = 0.640), and because of the lack of significant heterogeneity (*I*^*2*^ = 0%, *P* = 0.860), a fixed effects model was used (Fig. 4a). The RFS outcomes were reported in 3 studies, which included a total of 1172 gastric cancer patients. The pooled results suggested that the RFS outcomes were similar between the RG and LG groups (HR = 0.91, 95% CI 0.69 ~ 1.21, *P* = 0.530). The analysis had no obvious heterogeneity (*I*^*2*^ = 0%, *P* = 0.910) using a fixed effects model (Fig. 4b). Five studies reported recurrence rates. The pooled results showed no significant difference in the recurrence rate between the two groups (OR = 0.90, 95% CI 0.67 ~ 1.21, *P* = 0.500), with no significant heterogeneity (*I*^*2*^ = 0%, *P* = 0.620) (Fig. 4c).

### Sensitivity analysis

**Fig. 5:**
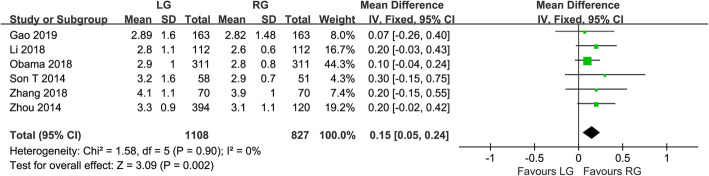
Forest plot of the sensitivity analysis for the time to first flatus

**Fig. 6:**
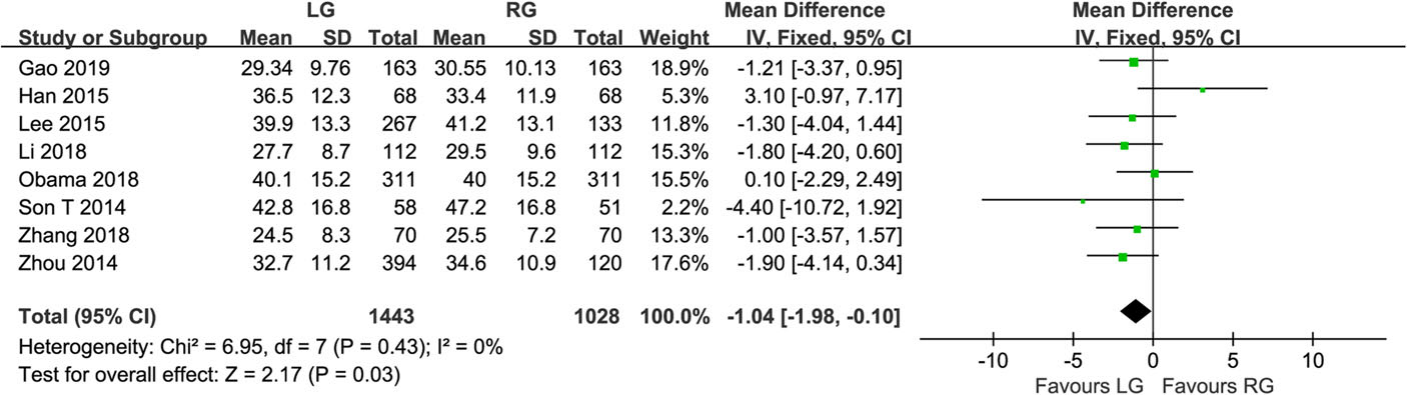
Forest plot of the sensitivity analysis for the number of retrieved lymph nodes

We conducted a sensitivity analysis for high-quality papers with more than 7 stars. In terms of the time to first flatus, the results showed that there was significant difference between the two techniques (WMD = 0.15, 95% CI 0.05 ~ 0.24, *P* = 0.002). The time to first flatus was shorter in RG than LG, with no significant heterogeneity (*I*^*2*^ = 0%, *P* = 0.900) (Fig. 5). In terms of the number of harvested lymph nodes, the results showed that the number of harvested lymph nodes was more in RG than LG (WMD = −1.04, 95% CI −1.98 ~ −0.10, *P* = 0.030), and there was no obvious heterogeneity (*I*^*2*^ = 0%, *P* = 0.430) (Fig. 6).

### Publication of bias

**Fig. 7:**
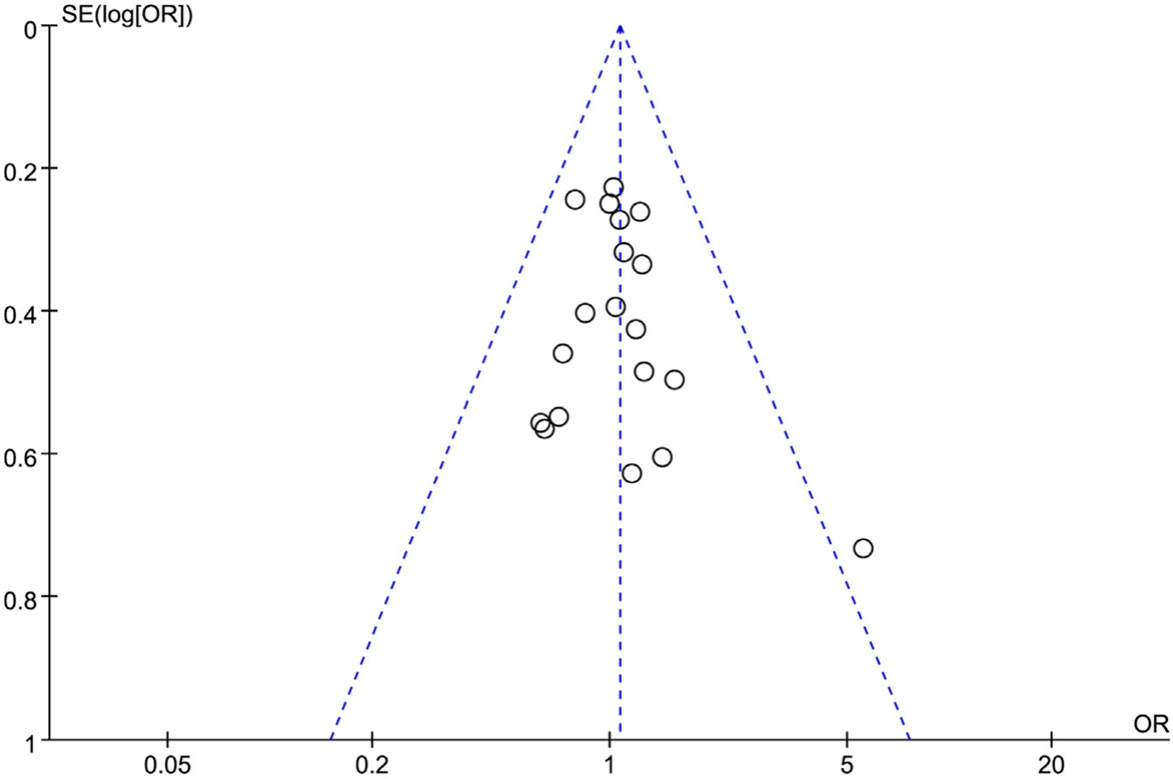
Funnel plot of the overall postoperative complications

A funnel plot of overall complications was utilized to evaluate publication bias. The bilaterally symmetrical funnel plot of overall complications showed that no evidence of publication bias was found (Fig. 7).

## Discussion

Radical gastrectomy with lymphadenectomy is regarded as gold standard of treatment for gastric cancer [40]. With the developing of minimally invasive techniques, MIS has gained a revolutionized application in gastrectomy. However, for gastric cancer, MIS experiences a controversy focusing on complication and mortality for a long time. MIS increases quality of life, but it should be ensured that this technique does not increase complication and mortality, especially the new technique-RG [41]. Many studies have compared the safety and short or long term efficacy of LG with open gastrectomy [42–46], but studies on RG have not been sufficient to show the effectiveness. We included 19 studies and performed a meta-analysis to explore and compare the clinical efficacy of RG and LG.

The results of meta-analysis suggested that RG was associated with longer operative time, compared with LG. On one hand, the reason might come from time of setting and docking the robotic arms, which resulted in a longer operative time [47]. Studies had shown that it took about 30 min to prepare for robotic surgery [48]. On the other hand, the difference of the experience of surgeons might cause a longer operative time. Previous research reported that the operative time for RG decreased between the initial RG and gastrectomies performed after experience had been gained [49–51]. Woo et al. [52] reported 236 cases of robotic gastrectomy and found that the mean operative time was reduced from 233 to 219 min when compared with the previous 100 cases. Song et al. [53] described that after 25 initial learning cases, the time for docking and setting up the robotic arm was shortened and kept stable, about 15 min. Therefore, docking times can be shortened after accumulation of greater experience. In addition, the learning curve for RG can increase the operative time. With the development of the Da Vinci robotic surgery system, more experience, and a shortened learning curve, can make the robot surgery more and short time.

Blood loss during minimally invasive gastrectomy mainly occurs during lymph node collection and is caused by vascular damage. The meta-analysis indicated that the blood loss was lower in RG than LG. The reason may be that robotic surgery has a high-definition visual field, eliminates hand tremors, and accurately reveals the small structure around the stomach, which helps surgeons better control bleeding in small blood vessels. Time to first flatus is a potential factor that should have an important impact on postoperative recovery. The results of meta-analysis suggested that there was a significant difference in time to first flatus, which was different from the results of previously published studies [17, 47, 54]. Therefore, in order to explore the reasons for the differences between the results, we conducted a sensitivity analysis according to the method described by Abraham et al. [55]. Abraham et al. stated that the results of combining high-quality non-randomized controlled trials were also convincing when comparing the short-term effects of surgery. The results of the sensitivity analysis showed that there was still a significant difference between the two groups, which indicated that the results of our study are reliable. The time to first flatus was shorter in RG than LG, which might be associated with the stable and flexible movements of the robotic arms, avoiding excessive traction on the tissue and accidental injury to the blood vessels, and less trauma to the patients [56]. In addition, the application of the concept of enhanced recovery after surgery (ERAS) in perioperative management may be another important reason for the significant difference in results. Zhang et al. [21] used this method to manage patients during the perioperative period and found that the time to first flatus in the RG group was significantly shorter than those in the LG group. Further prospective research is needed in order to confirm these advantages. However, the results of the meta-analysis showed that the potential factor could not cause the different postoperative hospital stay between the groups of RG and LG. There was no statistical difference between the two groups on hospital stay, but it seemed to prefer the RG.

The postoperative complication rate is an important indicator of the short-term outcome. This meta-analysis indicated that the incidence of overall complications in the group of RG was less than in the LG group, although no statistical difference. Regarding the mortality, analysis of the pooled data of the included studies suggested that mortality did not differ significantly between the two groups. According to these results, we believe that RG is safe and acceptable.

The result of tumor pathology is the key to evaluate the success of gastric cancer operation. This meta-analysis revealed that there was no significant difference in proximal margin and distal margin between the two groups. Radical gastric cancer surgery requires extensive lymph node dissection, which helps to more accurately assess the gastric cancer staging and prognosis of the patients. Regarding the number of harvested lymph nodes, analysis of the pooled data of the included studies revealed that the number of harvested lymph nodes was similar between the two groups, with no statistical difference. Our results were similar to the results of previously published studies [17, 47]. Recently, Guerrini et al. [54] published the largest meta-analysis of robotic versus laparoscopic gastrectomy for gastric cancer. The results showed that there was a significant difference in the number of harvested lymph nodes between the two groups, which was contrary to our results. Therefore, we conducted a sensitivity analysis by combining high-quality studies to explore the reasons for the opposite results. The results of the sensitivity analysis showed that there was a significant difference between the two groups. It was found that RG was associated with a significantly increased number of harvested lymph nodes, compared with LG. The main reason is that RG has three-dimensional imaging, a tremor filter, and an internal articulated EndoWrist with 7 degrees of freedom, which contribute to precise dissection and lymphadenectomy, especially the lymph nodes of the soft tissue around the gastric vessels [17]. Moreover, it may also be related to the continuous advancement of the robotic surgery system and the improvement of the proficiency of surgeons in its operation [57]. According to the standard of radical gastric cancer surgery, whether in the resection of the primary tumor or lymph node dissection, RG can achieve the goal of radical gastrectomy. However, large-scale and multi-center clinical randomized controlled trials are needed to provide more reliable evidence for clinical treatment in the future.

Because gastric cancer is a malignant tumor, the long-term follow-up oncological outcomes of gastric cancer patients were major concerns of surgeons. OS is a major oncologic outcome. In this meta-analysis, the OS was similar to that previously reported [58]. The pooled data of the included studies revealed no significant difference between the RG and LG groups in OS, RFS, and the recurrence rate without heterogeneity. These results showed that the two techniques had similar long-term oncologic outcomes. As far as we know, few meta-analyses had previously reported RFS with RG and LG. The RFS and recurrence rate results further demonstrated the comparability between RG and LG as far as long-term oncological outcomes in this meta-analysis. These results confirmed that in terms of oncologic outcomes, RG is a safe technique for the management of gastric cancer.

When considering these results in clinical application, several limitations need to be taken into account. First, our meta-analysis included a large number of patients, but all studies included for analysis were retrospective studies, and none were randomized controlled trials, which influence the quality of meta-analysis and result in publication bias. However, no significant publication bias was shown in this meta-analysis. Second, some studies did not describe HRs and SDs directly. These data were extracted from the survival curves, which could cause a potential source of bias. Third, most of included studies were from East Asian countries, and the data regarding Western countries was limited. The generalizability and applicability of these results were limited. These results must be interpreted with caution. Finally, we found that the heterogeneities of operative time, blood loss, and number of retrieved lymph nodes were all significant. These parameters could be influenced by the experience of surgeons.

## Conclusion

In conclusion, the results suggested that RG is as acceptable as LG in terms of short-term and long-term outcomes. Overall, our meta-analysis revealed that RG is an effective, safe, and promising approach in the treatment of gastric cancer, and makes up for the defects of laparoscopy, which can make patients have less trauma and quicker recovery. More randomized clinical trials are still essential to further indicate the value of the robotic surgery for gastric cancer.

## Supplementary Information

**Additional file 1.**

## Data Availability

The data in this manuscript are all provided in the tables and texts.

## Abbreviations

BMI: Body mass index
CI: Confidence interval
CNKI: China national knowledge infrastructure
D: Distal gastrectomy
EBL: Estimated blood loss
ERAS: Enhanced recovery after surgery
HR: Hazard ratios
LG: Laparoscopic gastrectomy
MIS: Minimally invasive surgery
NOS: Newcastle-Ottawa Scale
OR: Odds ratios
OS: Overall survival
P: Proximal gastrectomy
PPG: Pylorus-preserving gastrectomy
PRISMA: Preferred Reporting Items for Systematic Reviews and Meta-Analyses
R: Retrospectively collected data
RFS: Recurrence-free survival
RG: Robotic gastrectomy
SDs: Standard deviations
T: Total gastrectomy
WMD: Weighted mean difference
