# Supplementary material for: Robotic versus laparoscopic gastrectomy for gastric cancer: a systematic review and meta-analysis
Source: World J Surg Oncol. 2020 Nov 24;18:306. doi: 10.1186/s12957-020-02080-7 (PMC7688002; doi:10.1186/s12957-020-02080-7)
Supplement: Supplementary file 1 — Additional file 1. [file 12957_2020_2080_MOESM1_ESM.doc]

| **Section/topic** | **#** | **Checklist item** | **Reported on page #** |
| --- | --- | --- | --- |
| **TITLE** | | |  |
| Title | 1 | Robotic Versus Laparoscopic Gastrectomy for Gastric Cancer: a Systematic Review and Meta-Analysis | 1 |
| **ABSTRACT** | | |  |
| Structured summary | 2 | Background:To date,there is no clear conclusion whether RG can achieve an equal or even better surgical effect to LG.  Objective:To evaluate short- and long-term outcomes of robotic gastrectomy (RG) in gastric cancer patients to determine whether RG can replace laparoscopic gastrectomy (LG).  Data Sources:The Preferred Reporting Items for Systematic Reviews and Meta-Analyses statement was applied to perform the study.Pubmed,Cochrane Library,WanFang,CNKI and VIP databases were comprehensively searched for studies published before May 2020 that compared RG with LG.Only the studies in Chinese and English were included.Search terms included gastric cancer,gastric carcinoma, laparoscopic, robotic,and gastrectomy.  Study Selection:Clinical research comparing RG with LG for patients with gastric cancer,full-text article containing necessary data for statistical analysis,and the larger scale number publication or high quality publication were included.  Data Extraction: Independent extraction of articles by 2 authors using predefined data fields, including study quality indicators.  Data Synthesis:If the test of heterogeneity was high (I2>50% or P<0.05),a random-effect model was adopted.Otherwise,we used a fix effect model.A total of 19 studies including 7275 patients were included in the meta-analyses,of which 4598 patients were in the LG group and 2677 in the RG group.Compared with LG,RG was associated with longer operative time (WMD=−32.96 min; 95% CI:-42.08~-23.84, P<0.00001),less blood loss (WMD=28.66 ml; 95% CI: 18.59~38.73, P<0.00001),and shorter time to first flatus (WMD=0.16days; 95%CI:0.06~0.27, P=0.003).There was no significant difference between RG and LG in terms of the hospital stay (WMD=0.23days, 95 % CI:-0.53~0.98, P=0.56),overall postoperative complication (OR=1.07, 95 % CI:0.91~1.25, P=0.43),mortality (OR=0.67, 95% CI=0.24~1.90, P=0.45),the number of harvested lymph nodes (WMD=-0.96, 95% CI:-2.12~0.20, P=0.10),proximal resection margin (WMD=-0.10 cm,95% CI:-0.29~0.09, P=0.30),and distal resection margin (WMD=0.15cm,95% CI:-0.21~0.52, P=0.41).No significant differences were found between the two treatments in overall survival(OS) (HR=0.95, 95% CI:0.76~1.18; P=0.64), recurrence-free survival(RFS) (HR=0.91, 95% CI:0.69~1.21;P=0.53), and recurrence rate (OR=0.90, 95% CI:0.67~1.21; P=0.50).  Conclusions:The results of this study suggested that RG is as acceptable as LG in terms of short-term and long-term outcomes.RG can be performed as effectively and safely as LG. | 2-3 |
| **INTRODUCTION** | | |  |
| Rationale | 3 | In recent years,LG has been recognized for its advantages of MIS in the treatment of gastric cancer.Clinical trials comparing laparoscopic with open surgery have shown that laparoscopic radical gastrectomy has the same long-term effects as open radical gastrectomy.However,conventional laparoscopic surgery has also limitations of itself,including two-dimensional images,decreased sense of touch,amplification of hand tremor,et al.Recently,robot‐assisted surgery,an emerging technology,has been used to overcome the technical drawbacks of conventional laparoscopic surgery.Many studies have reported the safety and feasibility of RG,and are meaningful in highlighting the status of RG in the treatment of gastric cancer.However,these studies included small samples size,a single institution design and different appraise system of complications,which limited them to conclude objective result. | 4-5 |
| Objectives | 4 | To examine whether RG can achieve an equal or even better surgical effect to LG, we reviewed retrospective studies that assessed the clinical efficacy of RG compared with LG. | 5 |
| **METHODS** | | |  |
| Protocol and registration | 5 | Methods of the analysis and inclusion criteria were specified in advance and documented in a protocol. | 5 |
| Eligibility criteria | 6 | Types of studies: Retrospective studies studying the clinical efficacy of RG compared with LG. Only the Chinese and English studies that published before May 2020 were included.  Types of participants: Participants with gastric cancer were considered.Patients with gastrointestinal stromal tumors or benign gastric diseases were excluded from this review.  Types of intervention:Clinical research comparing RG with LG for patients with gastric cancer.  Types of outcome measures:Short-term outcomes:operative time,estimated blood loss (EBL),time to flatus,length of hospital stay,retrieved lymph nodes,proximal and distal margin distance,complications and mortality.Long‑term outcomes:OS,RFS and recurrence rate. | 5-7 |
| Information sources | 7 | Studies were identified by searching electronic databases and scanning reference lists of articles.Only the studies in Chinese and English were included.This search was applied to Pubmed (Before 2020), Cochrane Library (Before 2020),WanFang (Before 2020), CNKI (Before 2020) and VIP (Before 2020).The last search was run on 1 May 2020. | 5 |
| Search | 8 | In text: “We used the following search terms to search all databases:gastric cancer,gastric carcinoma, laparoscopic, robotic,and gastrectomy.  In appendix: Search strategy:Pubmed  ("stomach neoplasms"[MeSH Terms] OR ("stomach"[All Fields] AND "neoplasms"[All Fields]) OR "stomach neoplasms"[All Fields] OR ("gastric"[All Fields] AND "cancer"[All Fields]) OR "gastric cancer"[All Fields] OR (("gastrics"[All Fields] OR "stomach"[MeSH Terms] OR "stomach"[All Fields] OR "gastric"[All Fields]) AND ("carcinoma"[MeSH Terms] OR "carcinoma"[All Fields] OR "carcinomas"[All Fields] OR "carcinoma s"[All Fields]))) AND ("laparoscopes"[MeSH Terms] OR "laparoscopes"[All Fields] OR "laparoscope"[All Fields] OR "laparoscopical"[All Fields] OR "laparoscopically"[All Fields] OR "laparoscopics"[All Fields] OR "laparoscopy"[MeSH Terms] OR "laparoscopy"[All Fields] OR "laparoscopic"[All Fields]) AND ("robot"[All Fields] OR "robot s"[All Fields] OR "robotically"[All Fields] OR "robotics"[MeSH Terms] OR "robotics"[All Fields] OR "robotic"[All Fields] OR "robotization"[All Fields] OR "robotized"[All Fields] OR "robots"[All Fields]) AND ("gastrectomy"[MeSH Terms] OR "gastrectomy"[All Fields] OR "gastrectomies"[All Fields]) | 5 |
| Study selection | 9 | Eligibility assessment was performed independently in an unblinded standardized manner by 2 reviewers.Disagreements between reviewers were resolved by consensus. | 6 |
| Data collection process | 10 | Two authors independently and carefully reviewed and extracted the effective data from all included studies according to the inclusion and exclusion criteria,and checked the results again.If there was a disagreement,the controversial results were resolved by further discussion, and a final decision was made. | 6 |
| Data items | 11 | Information was extracted from each included trial on: (1) Main characteristics of included studies (including first author,publication year,country,study design,sample size,age,body mass index(BMI),and extent of resection), and the trial’s inclusion and exclusion criteria; (2) type of intervention (including type of surgery and extent of resection; (3) type of outcome measure (including estimated blood loss (EBL),time to flatus,retrieved lymph nodes,operative time,length of hospital stay,proximal and distal margin distance,complications,mortality,OS,RFS and recurrence rate). | 6-7 |
| Risk of bias in individual studies | 12 | To ascertain the validity of eligible clinical trials, pairs of reviewers working independently and with adequate reliability used the Newcastle-Ottawa Scale to estimate the quality of included studies(Representativeness of the exposed cohort,Selection of the non exposed cohort,Ascertainment of exposure,Demonstration that outcome of interest was not present at start of study,Assessment of outcome,Was follow-up long enough for outcomes to occur,Adequacy of follow up of cohorts).Scores range from 0 to 9 stars：studies with a score higher than or equal to 7 were considered to be high-quality and were included in the meta-analysis. | 7 |
| Summary measures | 13 | Continuous variables were assessed using weighted mean difference (WMD) and a 95% confidence interval (CI) and dichotomous variables using odds ratios (OR) with a 95% CI.The survival data,such as OS and RFS,were assessed using the hazard ratios (HR) and a 95% CI.If the test of heterogeneity was high (I2>50% or P<0.05),a random-effect model was adopted.Otherwise,we used a fix effect model. | 7 |
| Synthesis of results | 14 | The meta-analysis was performed by using the Review Manager 5.3 software (Cochrane Collaboration, Oxford, UK).Continuous variables were assessed using weighted mean difference (WMD) and a 95% confidence interval (CI) and dichotomous variables using odds ratios (OR) with a 95% CI.The survival data,such as OS and RFS,were assessed using the hazard ratios (HR) and a 95% CI.The I2 statistics was utilized to evaluate the heterogeneity.I2<25%, 25%≤I2≤50% and I2>50% were regarded as low, moderate and high heterogeneity.If the test of heterogeneity was high (I2>50% or P<0.05),a random-effect model was adopted.Otherwise,we used a fix effect model.Operation time(I2=94%),Estimated blood loss(I2=81%),Retrieved lymph nodes(I2=83%),Proximal margin(I2=28%),Distal margin(I2=59%),Time to first flatus(I2=65%),Hospital stay(I2=93%),Overall complications(I2=0%),Mortality(I2=0%),Overall survival(I2=0%),Recurrence-free survival(I2=0%),Recurrence rate(I2=0%). | 7,14 |

Page 1 of 2

| **Section/topic** | **#** | **Checklist item** | **Reported on page #** |
| --- | --- | --- | --- |
| Risk of bias across studies | 15 | For each trial we plotted the effect by the inverse of its standard error. The symmetry of such ‘funnel plots’ was assessed visually.Funnel plot was utilized to evaluate the potential publication of bias according to the overall complication. | 8 |
| Additional analyses | 16 | We conducted a sensitivity analysis for high-quality papers with more than 7 stars. | 14 |
| **RESULTS** | | |  |
| Study selection | 17 | A total of 19 studies were included.The search of Pubmed,Cochrane Library,WanFang,CNKI and VIP databases provided a total of 430 potential articles.After removing 66 duplicates,246 studies excluded by carefully reading the titles and abstracts because it was a review,letter,conference report,comment,case report or animal experimental study.118 potential articles were thoroughly evaluated through full text articles,and 80 articles were excluded because of failure to meet inclusion criteria(not comparing RG and LG:45,study including non-gastric cancer cases or benign gastric diseases:13,without necessary data for statistical analysis:11,repeated reported between authors and institution:7,no full text:4).19 of the remaining 38 articles were excluded because the score was less than 7. Finally,a total of 19 retrospective studies were included in the final meta-analysis according to inclusion and exclusion criteria.No unpublished relevant studies were obtained.  See flow diagram in fig 1. | 8 |
| Study characteristics | 18 | 19 studies with a total of 7275 patients, of which 4598 patients were in the LG group and 2677 in the RG group,were involved.14 of the included studies were published in English,and 5 published in Chinese.Among the 19 studies,13 were from China,4 from Korea and 2 from Japan.The basic characteristics of the included studies are listed in Table 1. | 8-9 |
| Risk of bias within studies | 19 | The evaluation of quality according to the NOS is shown in Table 2. NOS shows that 6 out of the 19 studies observed had 9 stars,2 had 8 stars,and 11 had 7 stars. | 8-9,11 |
| Results of individual studies | 20 | Fig. 2,Fig. 3,Fig. 4 and Table 3 shows the results of meta-analysis for short-term and long-term outcomes. | 11,14 |
| Synthesis of results | 21 | Meta-analysis revealed that the operative time was longer for RG than for LG (WMD=−32.96 min; 95% CI:-42.08~-23.84, P<0.00001,I2=94%).The meta-analysis showed that the EBL was lower in RG than LG (WMD=28.66 ml ;95% CI: 18.59~38.73, P<0.00001,I2=81%).Pooled analysis showed that the number of days to first flatus of RG was shorter than LG,with a high heterogeneity (WMD=0.16days;95% CI:0.06~0.27, P=0.003,I2=65%).The pooled results showed no difference in hospital stay between the RG and LG groups (WMD=0.23days, 95 % CI:-0.53~0.98, P=0.56,I2=93%).All 19 studies present the overall postoperative complication.Analysis of the index revealed no significant difference between the groups of RG and LG (OR=1.07, 95 % CI:0.91~1.25, P=0.43,I2=0%).Although no significant difference could be found in mortality between the two techniques,the pooled result revealed that LG group had a higher mortality than RG group (OR=0.67, 95% CI=0.24~1.90, P=0.45,I2=0%).Analysis of the index revealed that harvested lymph nodes were similar between the groups of RG and LG (WMD=-0.96, 95% CI:-2.12~0.20, P=0.10,I2=83%).The proximal margin was not significantly different between the two groups (WMD=-0.10 cm,95% CI:-0.29~0.09, P=0.30,I2=28%).In terms of the distal margin,the difference between the two groups was not also significant (WMD=0.15cm,95% CI:-0.21~0.52, P=0.41,I2=59%).The OS outcomes were recorded in 6 studies.Pooled analysis indicated no significant difference between the two techniques (HR=0.95,95% CI:0.76~1.18; P=0.64,I2=0%).The pooled results suggested that the RFS outcomes were similar between the RG and LG groups (HR=0.91,95% CI:0.69~1.21;P=0.53,I2=0%).The pooled results showed no significant difference in recurrence rate between the two groups (OR=0.90,95% CI:0.67~1.21; P=0.50,I2=0%). | 11-14 |
| Risk of bias across studies | 22 | A funnel plot of overall complications was utilized to evaluate publication bias.The bilaterally symmetrical funnel plot of overall complications shown that no evidence of publication bias was found. | 15 |
| Additional analysis | 23 | In terms of the time to first flatus,the results showed that there was significant difference between the two techniques (WMD=0.15days;95% CI:0.05~0.24, P=0.002). The time to first flatus was shorter in RG than LG,with no significant heterogeneity (I2=0%,P=0.900).In terms of the number of harvested lymph nodes,the results showed that the number of harvested lymph nodes was more in RG than LG (WMD=-1.04, 95% CI:-1.98~-0.10, P=0.030),and there was no obvious heterogeneity (I2=0%,P=0.430). | 14 |
| **DISCUSSION** | | |  |
| Summary of evidence | 24 | The results of meta-analysis suggested that RG was associated with longer operative time, compared with LG(WMD=−32.96 min; 95% CI:-42.08~-23.84, P<0.00001,I2=94%).On one hand,the reason might come from time of setting and docking the robotic arms,which results in a longer operative time .46Studies had shown that it took about 30 minutes to prepare for robotic surgery.47On the other hand,the difference of surgeons’ experience might cause a longer operative time.Previous research reported that the operative time for RG decreased between the initial RG and gastrectomies performed after experience had been gained.The meta-analysis indicated that the blood loss was lower in RG than in LG(WMD=28.66 ml ;95% CI: 18.59~38.73, P<0.00001,I2=81%).The reason might be that robotic surgery has a high-definition visual field, eliminates hand tremors and accurately reveals the small structure around the stomach,which help surgeons better control bleeding in small blood vessels.  The results of meta–analysis suggested that there was significant difference in time to first flatus, and the time to first flatus was shorter in RG than in LG(WMD=0.16days;95% CI:0.06~0.27, P=0.003,I2=65%).The reason may be associated with minimal invasion and small stress response of RG.In addition,application of the concept of enhanced recovery after surgery (ERAS) in perioperative management may be another important reason for the significant difference in results.The results of meta-analysis showed that the potential factor could not cause the different postoperative hospital stay between the groups of RG and LG(WMD=0.23days, 95 % CI:-0.53~0.98, P=0.56,I2=93%).This meta-analysis indicated that the incidence of overall complications in the group of RG was less than in LG group, although no statistical difference(OR=1.07, 95 % CI:0.91~1.25, P=0.43,I2=0%).Regarding the mortality, analysis of the pooled data of the included studies suggested that mortality did not differ significantly between the two groups (OR=0.67, 95% CI=0.24~1.90, P=0.45,I2=0%).According to these results,we believe that RG is safe and acceptable.This meta analysis revealed that there were no significant difference in proximal margin and distal margin between the two groups(WMD=-0.10 cm,95% CI:-0.29~0.09, P=0.30,I2=28%) (WMD=0.15cm,95% CI:-0.21~0.52, P=0.41,I2=59%).Regarding the number of harvested lymph nodes, analysis of the pooled data of the included studies revealed that the number of harvested lymph nodes in the group of RG was more than in LG group, although no statistical difference(WMD=-0.96, 95% CI:-2.12~0.20, P=0.10,I2=83%).The results of the sensitivity analysis showed that there was significant difference between the two groups.It was found that RG was associated with a significantly increased number of harvested lymph nodes, compared with LG.The main reason is that RG has three-dimensional imaging,a tremor filter,and an internal articulated EndoWrist with 7°of freedom,which contribute to precise dissection and lymphadenectomy,especially the lymph nodes of the soft tissue around the gastric vessels.Moreover,it may also be related to the continuous advancement of robotic surgery system and the improvement of the surgeon's proficiency in its operation.The pooled data of the included studies revealed no significant difference between the RG and LG groups in OS, RFS, and the recurrence rate without heterogeneity(HR=0.95,95% CI:0.76~1.18; P=0.64,I2=0%) (HR=0.91,95% CI:0.69~1.21;P=0.53,I2=0%) (OR=0.90,95% CI:0.67~1.21; P=0.50,I2=0%). These results show that the two techniques have similar long-term oncologic outcomes. | 15-19 |
| Limitations | 25 | Outcome level: We found that the heterogeneities of operative time,blood loss,and number of retrieved lymph nodes were all significant.These parameters can be influenced by surgeons’ experience.  Study and review level:First,our meta-analysis included a large number of patients,but all studies included for analysis were retrospective studies,and none were randomized controlled trials, which influence the quality of meta-analysis and result in publication bias.Second,some studies did not describe HRs and SEs directly.These data were extracted from the survival curves, which could cause a potential source of bias.Finally,most of included studies were from East Asia East Asian countries, and the data regarding Western countries are limited. The generalizability and applicability of these results are limited. | 20 |
| Conclusions | 26 | Implications for practice:The results suggested that RG is as acceptable as LG in terms of short-term and long-term outcomes.Our meta-analysis revealed that RG is an effective,safe and promising approach in treatment of gastric cancer,and make up for the defects of laparoscopy,which make patients have less trauma and quick recovery.  Implications for research:More randomized clinical trials are still essential to further indicate the value of the robotic surgery for gastric cancer. | 20 |
| **FUNDING** | | |  |
| Funding | 27 | This study was supported by the Natural Science Foundation of Shanghai (Grant No. 17ZR1439300); the Scientific Research Program of Shanghai Municipal Commission of Health and Family Planning (Grant No. 201640269);National Natural Science Foundation of China (Grant No. 81772955).  Role of funding source: The funders played a role in study design. | 21 |

*From:*  Moher D, Liberati A, Tetzlaff J, Altman DG, The PRISMA Group (2009). Preferred Reporting Items for Systematic Reviews and Meta-Analyses: The PRISMA Statement. PLoS Med 6(7): e1000097. doi:10.1371/journal.pmed1000097

For more information, visit: **www.prisma-statement.org**.

Page 2 of 2
